# Understanding cognitive functioning in glioma patients: The relevance of IDH‐mutation status and functional connectivity

**DOI:** 10.1002/brb3.1204

**Published:** 2019-02-26

**Authors:** Jolanda Derks, Shanna Kulik, Pieter Wesseling, Tianne Numan, Arjan Hillebrand, Edwin van Dellen, Philip C. de Witt Hamer, Jeroen J. G. Geurts, Jaap C. Reijneveld, Cornelis J. Stam, Martin Klein, Linda Douw

**Affiliations:** ^1^ Department of Anatomy & Neurosciences VU University Medical Center Amsterdam The Netherlands; ^2^ VUmc CCA Brain Tumor Center Amsterdam Amsterdam The Netherlands; ^3^ Department of Pathology VU University Medical Center Amsterdam The Netherlands; ^4^ Department of Pathology, Princess Máxima Center for Pediatric Oncology University Medical Center Utrecht Utrecht The Netherlands; ^5^ Department of Clinical Neurophysiology and MEG Center VU University Medical Center Amsterdam The Netherlands; ^6^ Department of Psychiatry University Medical Center Utrecht Utrecht The Netherlands; ^7^ Brain Center Rudolf Magnus Utrecht The Netherlands; ^8^ Department of Neurosurgery Neuroscience Campus Amsterdam, VU University Medical Center Amsterdam The Netherlands; ^9^ Department of Neurology Neuroscience Campus Amsterdam, VU University Medical Center Amsterdam The Netherlands; ^10^ Department of Medical Psychology VU University Medical Center Amsterdam The Netherlands; ^11^ Athinoula A. Martinos Center for Biomedical Imaging/Massachusetts General Hospital Charlestown Massachusetts

**Keywords:** cognition, diffuse glioma, isocitrate dehydrogenase, magnetoencephalography

## Abstract

**Introduction:**

Cognitive deficits occur frequently in diffuse glioma patients, but are limitedly understood. An important marker for survival in these patients is isocitrate dehydrogenase (IDH) mutation (IDH‐mut). Patients with IDH‐mut glioma have a better prognosis but more often suffer from epilepsy than patients with IDH‐wildtype (IDH‐wt) glioma, who are generally older and more often have cognitive deficits. We investigated whether global brain functional connectivity differs between patients with IDH‐mut and IDH‐wt glioma, and whether this measure reflects variations in cognitive functioning in these subpopulations beyond the associated differences in age and presence of epilepsy.

**Methods:**

We recorded magnetoencephalography and tested cognitive functioning in 54 diffuse glioma patients (31 IDH‐mut, 23 IDH‐wt). Global functional connectivity between 78 atlas regions spanning the entire cortex was calculated in two frequency bands (theta and alpha). Group differences in global functional connectivity were tested, as was their association with cognitive functioning, controlling for age, education, and presence of epilepsy.

**Results:**

Patients with IDH‐wt glioma had lower functional connectivity in the alpha band than patients with IDH‐mut glioma (*p = *0.040, corrected for age and presence of epilepsy). Lower alpha band functional connectivity was associated with poorer cognitive performance (*p* < 0.034), corrected for age, education, and presence of epilepsy.

**Conclusion:**

Global functional connectivity is lower in patients with IDH‐wt diffuse glioma compared to patients with IDH‐mut diffuse glioma. Moreover, having lower functional alpha connectivity relates to poorer cognitive performance in patients with diffuse glioma, regardless of age, education, and presence of epilepsy.

## 1 INTRODUCTION

Diffuse gliomas are characterized by poor survival and cognitive deficits (Taphoorn & Klein, 2004). An important marker for survival in patient with diffuse glioma is isocitrate dehydrogenase (IDH) mutation (IDH‐mut), which is associated with better prognosis but higher epilepsy prevalence (Chen et al., 2017; Yan et al., 2009). Patients with wildtype glioma (IDH‐wt) are generally older and more often have cognitive deficits (Wefel, Noll, Rao, & Cahill, 2016), even though they less often have epileptic seizures that are generally linked to poorer cognitive functioning (Chen et al., 2017; Klein, Engelberts, et al., 2003). Although the relevance of IDH status for survival is relatively straightforward (Yan et al., 2009), the higher occurrence of cognitive deficits at diagnosis in patients with IDH‐wt glioma as compared to patients with IDH‐mut glioma is incompletely understood. This is in part because of the mentioned group differences in age and prevalence of epilepsy. IDH‐wt‐related cognitive deficits have been linked to deterioration in cortical thickness networks (Kesler, Noll, Cahill, Rao, & Wefel, 2017). However, cortical thickness is highly age‐dependent (Thambisetty et al., 2010), indicating that age differences between IDH‐mut and IDH‐wt patients may obfuscate how much variance in this brain correlate of cognition is explained by the mutation itself.

Regardless of IDH‐mutation status, cognitive deficits in glioma patients are associated with altered brain functional connectivity. Functional connections are statistical correlations between regional activity as for instance measured with magnetoencephalography (MEG) (Bartolomei et al., 2006; Bosma et al., 2009, 2008; Carbo et al., 2017; van Dellen et al., 2012, 2013, 2014; Derks, Reijneveld, & Douw, 2014; Douw et al., 2010, 2008; Guggisberg et al., 2008; Tarapore et al., 2012). Cognitive variation, and presence and frequency of epilepsy in glioma patients, has been related to altered theta (4–8 Hz) (Bosma et al., 2008; van Dellen et al., 2012; Douw et al., 2010) and alpha (8–13 Hz) (Bosma et al., 2009; Carbo et al., 2017; van Dellen et al., 2013) band functional connectivity.

We performed MEG and cognitive testing in a cohort of de novo glioma patients and investigated whether theta and alpha global brain functional connectivity differed according to IDH‐mutation status and whether this measure reflects cognitive functioning in these subpopulations beyond differences in age and presence of epilepsy. We expected lower functional connectivity to relate to poorer cognitive functioning (when correcting for age and presence of epilepsy), and thus functional connectivity to be lower in IDH‐wt patients as compared to IDH‐mut patients.

## 2 METHODS

### 2.1 Patients

Patients visiting the VUmc CCA Brain Tumor Center Amsterdam between 2010 and 2017 with suspected diffuse glioma were eligible to participate. Part of this patient cohort has been reported on before (Carbo et al., 2017; van Dellen et al., 2014, 2012; Derks et al., 2018). Inclusion criteria were (a) age over 17 years and (b) ability to participate in neuropsychological testing. After testing and MEG recording, all patients were diagnosed with World Health Organization (WHO) grade II, III, or IV diffuse glioma according to the WHO 2007 classification (Louis, Ohgaki, Wiestler, & Cavenee, 2007). Patients with previous craniotomies or neurological/psychiatric comorbidities were not able to participate in this study. Information on the presence of epilepsy, use of anti‐epileptic drugs, use of dexamethasone, and Karnofsky performance status (KPS) was collected (Karnofsky, Abelmann, & Craver, 1948). Level of education was gathered based on a commonly used Dutch scale for highest obtained educational degree, which ranges from level 1 (not completed primary education) to level 7 (academic degree) (Verhage, 1964). Tumors were manually drawn on 3D anatomical magnetic resonance imaging (MRI) images, slice by slice (LD), using both contrast‐enhanced T1‐weighted and FLAIR images. Tumor volume was assessed by calculating the volume of the voxels containing tumor. The ethical review board of the VU University Medical Center approved this study, and all patients gave written informed consent before participation.

### 2.2 IDH‐mutation status

IDH‐mutation status was assessed with immunohistochemistry on formalin‐fixed paraffin‐embedded tissue according to IDH‐mutation diagnostic routine, identifying 90% of all IDH‐mutant diffuse gliomas (Ichimura, Narita, & Hawkins, 2015). Diagnostic routine included detection of *IDH1* by IDH1R132H antibody (mouse monoclonal, clone H09; Dianova GmbH, Hamburg, Germany) using a Ventana Benchmark ULTRA (Roche Diagnostics, Mannheim, Germany). Pretreatment was done with Cell Conditioner 1 for 24 min followed by primary antibody incubation diluted (1:1,250) in background reducing antibody diluent (DAKO, Glostrup, Denmark) for 32 min. Immunostainings were visualized with Optiview DAB detection and Optiview amplification (Roche/Ventana Medical Systems, Tucson, AZ, USA). Tissue was counterstained with hematoxylin.

### 2.3 Cognitive functioning

Cognitive functioning was extensively measured preoperatively (Douw et al., 2009; Klein et al., 2002; Klein, Postma, et al., 2003; Taphoorn & Klein, 2004). As measures of cognitive performance, we included the sum score of the five trials, and the delayed recall score of the Rey Auditory Verbal Learning Test (RAVLT) (Rey, 1958) (verbal memory), the time measured on part C of the Concept Shifting Test (van der Elst, van Boxtel, van Breukelen, & Jolles, 2006a) (executive functioning and psychomotor speed), the duration of the last and most difficult assessment of the Memory Comparison Test (Brand & Jolles, 1987) (working memory), the score on the verbal version of the Letter Digit Substitution Test (van der Elst, van Boxtel, van Breukelen, & Jolles, 2006b) (information processing speed and psychomotor speed), the time of the color–word card of the Stroop Color Word Test (Stroop, 1935) (attentional functioning), and by the number of words generated during the Categorical Word Fluency test (Luteijn & van der Ploeg, 1983) (executive functioning).

### 2.4 Magnetoencephalography

Participants underwent MEG recording before neurosurgical intervention, and/or start of any radio‐ or chemotherapy (as described previously (Carbo et al., 2017; van Dellen et al., 2013, 2014)). In brief, an eyes‐closed resting state recording of 5 min in a magnetically shielded room (Vacuum Schmelze GmbH, Hanua, Germany) with a 306 channel MEG system (Elekta Neuromag Oy, Helsinki, Finland) was acquired. Data were sampled at 1,250 Hz, and a high‐pass filter (0.1 Hz) and anti‐aliasing filter (410 Hz) were employed online. Malfunctioning channels were excluded after visual inspection (JD, SK, TN, LD) of the neurophysiological signals after applying the extended Signal Space Separation method (xSSS) (van Klink et al., 2017). The removal of artefacts was done offline with the temporal extension of SSS in MaxFilter software (Elekta Neuromag Oy, version 2.2.15) (Taulu & Hari, 2009; Taulu & Simola, 2006), and then visually inspected for quality. For coregistration of MEG with participants’ MRI, the outline of the scalp and four or five head localization coils were digitized using a 3D digitizer (3Space Fastrak, Polhemus, Colchester, VT, USA) and matched to the MRI scalp surface. The coregistered MRI was then spatially normalized to a template MRI, and, using the Automated Anatomical Labeling (AAL) atlas (Tzourio‐Mazoyer et al., 2002), the centroid voxels (Hillebrand et al., 2016) in the 78 cortical regions (Gong et al., 2009) were selected for further analyses after inverse transformation to the patient's coregistered MRI. A scalar beamformer implementation (Elekta Neuromag Oy, version 2.1.28) was used to reconstruct broadband (0.5–48 Hz) time series of neuronal activity for these centroids (Hillebrand, Barnes, Bosboom, Berendse, & Stam, 2012). For each patient, 60 consecutive epochs of 3.27 s (4,096 samples) were used to extract theta and alpha time series. These were obtained by digitally filtering the selected epochs using a fast Fourier transform, after which all bins outside the passbands were made zero, and an inverse Fourier transform was performed. Theta and alpha powers were calculated, relative to the power in the broadband signal.

### 2.5 Global functional connectivity

Frequency band‐specific functional connectivity between reconstructed time series of each pair of atlas regions was assessed with the phase lag index (PLI) implemented in Matlab (version R2012.a, Mathworks, Natick, MA, USA). The PLI measures synchronization by using the asymmetry of the distribution of phase differences (Δφ) between two time series (Stam, Nolte, & Daffertshofer, 2007): PLI = |<sign[sin(Δφ(t_k_))]>|. The phase difference is defined in the interval [−π,π], <> denotes the mean value, tk is the sample index, and || indicates the absolute value. The PLI ranges between 0 and 1, with values closer to 0 indicating lower synchrony between two regions and values closer to 1 indicating higher synchrony. The PLI is minimally affected by volume conduction and field spread because the PLI only takes nonzero phase lag between two time series into account. The PLI was estimated between all pairs of regions for each epoch, forming a 78 × 78 matrix of functional connections. The 78 × 78 functional connectivity matrix was then averaged over epochs to yield a single measure of global functional connectivity per subject and per frequency band. A Box–Cox transformation (lambda = −5) (Box & Cox, 1964) was performed on global functional connectivity measures to ensure normality of the data; the transformed data were used in all statistical analyses.

### 2.6 Statistical analyses

Statistical analyses were performed using IBM SPSS Statistics for Windows (version 22.0.0.0 IBM Corp., Armonk, NY, USA). Group differences between patients with IDH‐mut and IDH‐wt glioma were investigated with Student's *t* tests for independent samples (age), Mann–Whitney *U* tests (tumor volume and education) and exact chi‐square tests (sex, WHO grade, tumor lateralization (excluding bilateral tumors), frontal versus non‐frontal tumor localization, presence of epilepsy, anti‐epileptic drug use, use of dexamethasone, and KPS dichotomized into ≤80 and 90–100 (Derks et al., 2017)). Group differences in cognitive functioning were established with (seven) linear regression models for each cognitive test described above, corrected for age, level of education, and presence of epilepsy. A log transformation was performed on the raw test scores of the Concept Shifting Test, the Memory Comparison Test, and of the Stroop Color Word Test to adhere to the assumption of normally distributed standardized residuals in linear regression analyses. The transformed data were used in all following statistical tests.

Group differences in theta and alpha functional connectivity (dependent variable) between IDH subgroups (independent variable) were computed with linear regression models to account for confounding variables (age and presence of epilepsy). In case of significant results, group differences in frequency‐specific relative power were investigated with linear regression as well, to ascertain that connectivity differences were not driven by differences in relative power.

In case of significant differences in functional connectivity between the subgroups, associations between functional connectivity and cognitive functioning were assessed. Linear regression models were computed for each cognitive test, with (log transformed) test score as the dependent variable and functional connectivity, age, presence of epilepsy, and level of education as independent variables. In addition, tumor volume was also added to these models, since tumor volume is possibly associated with cognitive functioning, particularly in patients with IDH‐wt glioma (Kesler et al., 2017).

Post hoc analyses included testing for the possible confounding effect of dexamethasone use, which may affect cognition (Kostaras, Cusano, Kline, Roa, & Easaw, 2014). First, differences in cognitive performance according to dexamethasone use were tested with seven linear regression models corrected for age, presence of epilepsy, and education. Next, a Student's *t* test was performed to test for differences in alpha functional connectivity according to dexamethasone use. Furthermore, histological WHO grade may confound functional connectivity changes beyond IDH‐mutation status. Therefore, we tested for differences in alpha functional connectivity between grade II/III and grade IV IDH‐wt patients with linear regression controlling for age and presence of epilepsy, as this subgroup had enough variation in WHO grade to do statistical testing.

All linear regression analyses met the criteria of normally distributed standardized residuals and homoscedasticity by visual inspection. A *p*‐value lower than 0.05 was considered significant.

## 3 RESULTS

### 3.1 Patient characteristics

In total, 54 patients (seventeen females) participated, with a mean age of 45.17 (*SD* 15.22) years. Twenty‐three patients had IDH‐wt glioma, while 31 patients had IDH‐mut glioma (Table 1). As expected, age and the distribution of WHO grade significantly differed between the two groups, with IDH‐wt patients being older (*t*(52) = 3.592, *p* = 0.001), more often having WHO grade IV glioma (*χ* ^2^ (2, *n* = 54) = 16.517, *p < *0.001) and more often using dexamethasone (*χ* ^2^ (1, *n* = 50) = 6.603, *p* = 0.010). Patients with IDH‐mut glioma more often had epilepsy (*χ* ^2^ (1, *n* = 54) = 8.693, *p* = 0.003) and more often used AEDs (*χ* ^2^ (1, *n* = 52) = 6.831, *p* = 0.009). There were no group differences in terms of level of education (*U* = 335, *p* = 0.912), sex (*χ* ^2^ (1, *n* = 54) = 0.151, *p* = 0.697), KPS (*χ* ^2^ (1, *n* = 51) = 0.053, *p* = 0.818), tumor lateralization (*χ* ^2^ (1, *n* = 52) = 0.171, *p* = 0.680), non‐frontal localization (*χ* ^2^ (1, *n* = 54) = 0.833, *p* = 0.361), or tumor volume (*U* = 256, *p* = 0.079).

**Table 1:** Patient characteristics

Patient characteristics	Glioma (*n = *54)	IDH‐wt (*n = *23)	IDH‐mut (*n = *31)	*p*‐value	Test statistic
Age (mean/*SD*)	45.17/15.22	52.97/17.23	39.38/10.48	0.001**	*t*(52) = 3.592
Sex (female/male)	18/36	7/16	11/20	0.697	*χ* ^2^ = 0.151
Education level (median/range)	6/2–7	6/2–7	6/2–7	0.912	*U* = 335
KPS (NA/<70/70–80/90–100)	3/1/12/39	2/1/4/16	1/0/8/22	0.818	*χ* ^2^ = 0.053
WHO grade (II/III/IV)	28/11/15	7/3/13	21/8/2	<0.001**	*χ* ^2^ = 16.517
Tumor hemisphere(left/right/bilateral)	30/22/2	14/9/0	16/13/2	0.680	*χ* ^2^ = 0.171
Tumor location (frontal/frontotemporal/temporal/parietotemporal/parietal/frontoparietal/occipital)	20/11/10/4/6/1/2	7/4/4/4/2/1/1	13/7/6/0/4/0/1	NA	NA
Epilepsy (yes/no)	43/11	14/9	29/2	0.010*	*χ* ^2^ = 6.627
Anti‐epileptic drug use (NA/yes/no)	2/30/12	1/13/9	1/27/3	0.009**	*χ* ^2^ = 6.831
Dexamethasone use (NA/yes/no)	4/8/42	0/7/16	4/1/26	0.010*	*χ* ^2^ = 6.603
Tumor volume cm^3^ (mean/*SD*)	57.70/42.96	48.06/38.37	64.85/45.35	0.079	*U* = 256

Note

Patient characteristics described for patients with IDH‐wt and patients with IDH‐mut glioma.

IDH‐wt, IDH‐wildtype; IDH‐mut, IDH‐mutation; *SD*, standard deviation; KPS, Karnofsky Performance Status; WHO, World Health Organization; NA, not available.

^*^ *p* < 0.05. ^**^ *p* < 0.01.

### 3.2 Cognitive functioning

Patients with IDH‐wt glioma performed significantly poorer on verbal memory (sum score RAVLT, *B* = 7.47, confidence interval (CI) = 1.39–13.56, *p* = 0.017; recall RAVLT, *B* = 3.23, CI = 1.46–5.01, *p* = 0.001; Table 2) compared to patients with IDH‐mut glioma, corrected for age, presence of epilepsy, and education. Patients with IDH‐wt glioma did not significantly differ from patients with IDH‐mut glioma on the other tests, but on average performed poorer on all tests (Table 2). Dexamethasone use was associated with performance on the Stroop Color Word Test (*B* = 0.171, CI = 0.005–0.337, *p* = 0.043), corrected for age, presence of epilepsy, and education.

**Table 2:** Linear regression models for IDH status and cognition

Cognitive test	IDH‐wt test score, mean (*SD*)	IDH‐mut test score, mean (*SD*)	IDH status *p*‐value	Age *p*‐value	Education *p*‐value	Presence of epilepsy *p*‐value	*B*‐value (CI)
Rey auditory verbal learning test sum of trials (*N* = 49)	40.5 (13.69)	49.93 (10.02)	0.017*	0.007**	<0.001**	0.301	7.472 (1.387 to 13.556)
Rey auditory verbal learning test recall (*N* = 49)	7.60 (3.58)	11.17 (2.65)	0.001**,^a^	0.032*	0.007*	0.242	3.233 (1.458 to 5.009)
Concept shifting test (*N* = 47)	38.91 (25.13)	30.82 (10.09)	0.792	<0.001**	0.002**	0.919	0.12 (−0.080 to 0.104)
Memory comparison test (*N* = 46)	74.90 (31.31)	62.63 (20.50)	0.197	0.849	0.040*	0.568	−0.068 (−0.172 to 0.037)
Categorical word fluency (*N* = 48)	21.42 (9.31)	24.45 (6.12)	0.123	0.885	0.001**	0.493	3.630 (−1.028 to 8.294)
Letter digit substitution test (*N* = 45)	53.82 (14.51)	57.11 (9.49)	0.574	0.094	0.006**	0.613	2.098 (−5.374 to 9.570)
Stroop color word test (*N* = 47)	120.23 (72.96)	97.17 (46.75)	0.755	0.001*	0.011*	0.490	−0.015 (−0.112 to 0.082)

Note

Linear regression models showing the difference in cognitive performance depending on IDH status, corrected for age, education and presence of epilepsy. Mean raw cognitive test scores are given for the IDH‐wt en IDH‐mut group. *B*‐values and 95% confidence intervals are displayed for IDH status.

CI, 95% confidence interval; IDH‐mut, IDH‐mutation; IDH‐wt, IDH‐wildtype; *SD*, standard deviation.

^*^ *p* < 0.05, ^**^ *p* < 0.01,

^a^ significant after false discovery rate correction.

### 3.3 Functional connectivity differs according to IDH status

**Figure 1:**
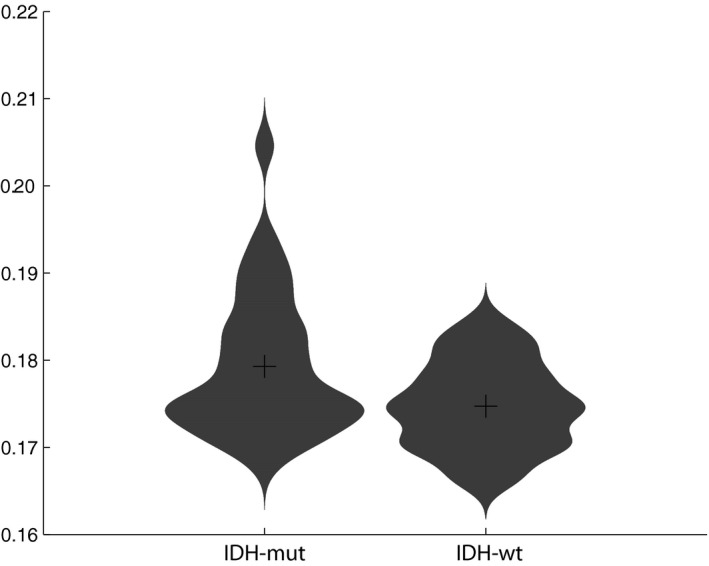
Violin plots showing alpha functional connectivity (before normalization) for IDH‐mut and IDH‐wt patients, the crosses within the plots indicate mean per group

Patients with IDH‐wt glioma had significantly lower alpha functional connectivity compared to patients with IDH‐mut glioma while controlling for age and presence of epilepsy (*B* = 138.209, CI = 6.575–269.842, *p* = 0.040, Figure 1). We did not control for tumor volume in this analysis because tumor volume was not significantly different between groups. These findings were specific to functional connectivity, as relative alpha power did not differ between groups (*B* = 0.005, CI = −0.006 to 0.016, *p* = 0.346), nor did alpha functional connectivity differ according to dexamethasone use (*t*(48) = 1.101, *p* = 0.276). Theta functional connectivity did not differ between groups (*B* = −14.925, CI = −49.732 to 19.883, *p* = 0.393).

As shown in Table 1, WHO grade varied in the IDH‐wt diffuse glioma cohort, which may relate to functional connectivity beyond mutation status. We therefore tested whether alpha functional connectivity differed between grade II/III and grade IV IDH‐wt patients with linear regression, while controlling for age and presence of epilepsy, which was not the case (*B* = −17.087, CI = −243.159 to 208.985, *p* = 0.876). These findings suggest that despite the presence of some level of malignant heterogeneity within patients with IDH‐wt glioma, WHO grade did not relate to alpha functional connectivity.

### 3.4 Functional connectivity explains cognitive variance across groups

**Figure 2:**
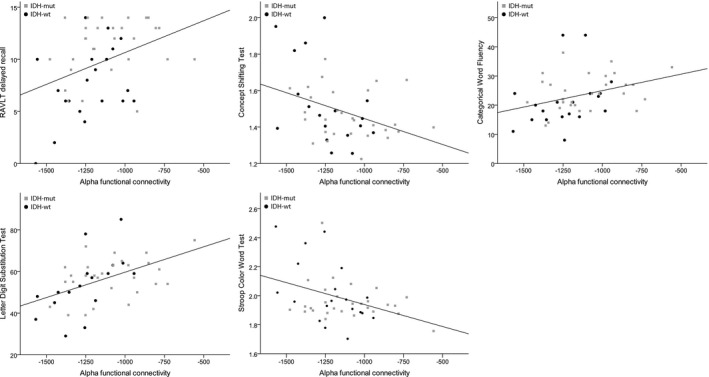
Associations between alpha functional connectivity and cognitive performance. Alpha functional connectivity (after normalization) is plotted on the x‐axis. The y‐axis reflects cognitive performance: RAVLT recall score (number of recalled words after delay), Concept Shifting Test (log transformed time to complete the interference condition), Categorical Word Fluency (number of animals listed in one minute), Letter Digit Substitution Test Score (number of letters completed after 90 s), Stroop Color Word Test (log transformed time to complete the interference condition). Black circles represent patients with IDH‐wt glioma, while gray squares represent patients with IDH‐mut glioma

In the entire cohort of patients, there were significant associations between alpha functional connectivity and cognitive test scores (after false discovery rate correction): Letter Digit Substitution Test (*B* = 0.020, CI = 0.007–0.033, *p* = 0.003), Stroop Color Word Test (*B* = −2.26e‐4, CI = −4.07e‐4 to −0.46e‐4 *p* = 0.015), RAVLT (recall, *B* = 0.005, CI = 0.001–0.01, *p* = 0.020), Concept Shifting Test (*B* = −1.92e‐4, CI = −3.66e‐4 to −0.18e‐4, *p* = 0.031), and the Categorical Word Fluency Test (*B* = 0.10, CI = 0.001–0.019, *p* = 0.034) while controlling for age, education, and presence of epilepsy (Figure 2, Table 3). All regression models indicate that lower functional connectivity in the alpha band is associated with poorer cognitive performance.

**Table 3:** Linear regression models for alpha functional connectivity and cognition

Cognitive test	Alpha functional connectivity *p*‐value	Age *p*‐value	Education *p*‐value	Presence of epilepsy *p*‐value	*B*‐value (CI)
Rey auditory verbal learning test sum of trials (*N* = 49)	0.062	0.002**	0.001**	0.902	0.012 (−0.001 to 0.025)
Rey auditory verbal learning test recall (*N* = 49)	0.020*,^a^	0.007**	0.050	0.888	0.005 (0.001 to 0.009)
Concept shifting test (*n = *47)	0.031*,^a^	<0.001**	0.002**	0.784	−1.92e‐4 (−3.66e‐4 to −0.18e‐4)
Memory comparison test (*n = *46)	0.067	0.739	0.062	0.994	−1.89e‐4 (−3.92e‐4 to 0.14e‐4)
Categorical word fluency (*n = *48)	0.034*,^a^	0.984	0.002**	0.986	0.100 (0.001 to 0.019)
Letter digit substitution test (*n = *45)	0.003**,^a^	0.103	0.008*	0.983	0.020 (0.007 to 0.033)
Stroop color word test (*n = *47)	0.015*,^a^	0.001*	0.010*	0.587	−2.26e‐4 (−4.07e‐4 to −0.46e‐4)

Note

Regression models were corrected for age, education and presence of epilepsy. *B*‐values and 95% confidence intervals are displayed for alpha functional connectivity.

CI, 95% confidence interval.

^*^ *p < *0.05, ^**^ *p* < 0.01,

^a^ significant after false discovery rate correction.

Addition of tumor volume to the model did not substantially modify these associations: Letter Digit Substitution Test score, (*B* = 0.019, CI = 0.006–0.032, *p* = 0.007), Stroop Color Word Test score (*B* = −2.08e‐4, CI = −3.92e‐4 to −0.24e‐4, *p* = 0.028), RAVLT (recall, *B* = 0.04, CI = −0.09e‐4 to 0.008, *p* = 0.50), Concept Shifting Test score (*B* = −1.62e‐4, CI = −3.38e‐4 to −0.14e‐4, *p* = 0.070), and Categorical Word Fluency Test (*B* = 0.010, CI = 3.23e‐4 to 0.019, *p* = 0.043).

As noted above, the use of dexamethasone was significantly related to performance on the Stroop Color Word Test (*B* = 0.171, CI = 0.005 to 0.337, *p* = 0.043). Therefore, we repeated the linear regression for the Stroop Color Word Test and alpha functional connectivity including dexamethasone use. Both alpha functional connectivity (*B* = −2.23e‐4 CI = −4.09e‐4 to −0.37e‐4, *p* = 0.020) and dexamethasone use (*B* = 0.164, CI = 0.008–0.321, *p* = 0.040) were independent predictors of Stroop Color Word Test performance.

## 4 DISCUSSION

Patients with IDH‐wt glioma have lower functional connectivity in the alpha band compared to patients with IDH‐mut glioma, even when controlling for age and presence of epilepsy. Moreover, lower functional connectivity is associated with poorer cognitive performance in the entire cohort.

Previous studies in glioma patients have amply shown whole brain alterations in functional connectivity compared to healthy controls (Derks et al., 2014), which are specifically relevant for cognition in theta (Bosma et al., 2008; van Dellen et al., 2012; Douw et al., 2010) and alpha (Bosma et al., 2009; Carbo et al., 2017; van Dellen et al., 2013) frequencies. The association between alpha band functional connectivity and cognition has been evidenced longitudinally as well (Carbo et al., 2017), showing increases in alpha band connectivity corresponding to improved cognitive functioning (van Dellen et al., 2013). Our current results build upon these studies, showing a positive correlation between alpha band functional connectivity and cognitive functioning, regardless of IDH‐mutation status.

These previous studies mainly reported on connectivity differences in glioma patients diagnosed according to the 2007 WHO classification, based on histopathology only (Derks et al., 2014; Louis et al., 2007). As the neuro‐oncology field has moved toward incorporating molecular markers like IDH‐mutation status (Louis, Ohgaki, Wiestler, & Cavenee, 2016), it is worth investigating the implications of this change for our understanding of cognition and its hypothetical neural correlates of connectivity and network topology. Although previous work has reported on connectivity differences between WHO grades (van Dellen et al., 2012), we did not find differences in functional connectivity between WHO grade II/III and grade IV patients within IDH‐wt patients. However, it is now well known that many WHO grade II and grade III IDH‐wt diffuse gliomas not only have molecular characteristics of, but also show clinical behavior of glioblastoma (WHO grade IV) (Cancer Genome Atlas Research Network et al., 2015). Our results suggest that molecular status, not WHO grade, drives cognitively relevant connectivity differences between diffuse glioma patients, although we were not able to statistically assess whether the same grade‐independence holds within the IDH‐mut patients. Larger cohorts are necessary to fully understand the apex of histopathological grade and molecular status in terms of functional connectivity.

Wefel and colleagues were the first to describe worse cognitive performance in patients with IDH‐wt glioma compared to patients with IDH‐mut glioma (Wefel et al., 2016). The same group investigated cortical thickness covariation as a neural correlate of this difference, showing that thickness covariation patterns indeed differ between subgroups and associate with cognition (Kesler et al., 2017). This provides a first insight into possible mechanisms underlying IDH‐mutation‐related cognitive differences and also suggests that tumor growth rate might contribute to cognitive problems (Klein, 2016; Wefel et al., 2016). However, the observed group differences in covarying cortical thickness were not corrected for age or presence of epilepsy. Since the cortex thins over time, regardless of the presence of glioma, as cognition also deteriorates, the reported results may have partly been due to normal aging (Thambisetty et al., 2010). Our results show alpha functional connectivity group differences irrespective of age and presence of epilepsy. Associations between alpha functional connectivity and cognitive functioning remained significant after controlling for these confounders. Our findings suggest that lower functional connectivity has a particular contribution to cognitive deterioration in IDH‐wt patients.

The fact that functional connectivity differed between IDH subgroups may reflect the impact of variable tumor growth rate on global connectivity (Klein, 2016; Wefel et al., 2016), while molecular mechanisms related to functional connectivity may also be at play. The overexpression of D‐2‐hydroxyglutarate in IDH‐mut glioma is of particular interest, as a preclinical study reported that this protein activates NMDA receptors specifically, thereby mimicking glutamatergic neuronal activation (Chen et al., 2017). The related increase in neuronal spiking could be the mechanism responsible for the higher incidence of epilepsy in patients with IDH‐mut glioma compared to patients with IDH‐wt glioma (Chen et al., 2017). Speculatively, the mimicry of glutamatergic neuronal signalling by D‐2‐hydroxyglutarate might also underlie alpha functional connectivity differences found in the current study, as glutamate plays an important role in the synchronization of neuronal oscillations (Angulo, Kozlov, Charpak, & Audinat, 2004; Fellin et al., 2004).

A limitation of this study is that we only tested gliomas for the R132 variant of *IDH1*, detecting approximately 90% of the IDH‐mutated gliomas (Ichimura et al., 2015). Hence, a few patients classified as having IDH‐wt glioma might actually have IDH‐mut glioma, meaning that we may have underestimated the group difference in global functional connectivity.

## 5 CONCLUSION

Alpha functional connectivity is lower in patients with IDH‐wt glioma as compared to patients with IDH‐mut glioma, regardless of age and presence of epilepsy. Moreover, alpha functional connectivity is positively associated with cognitive performance, irrespective of IDH status. These findings contribute to our understanding of cognitive functioning in patients with diffuse glioma in general, and cognitive deficits in patients with IDH‐wt glioma specifically.

## CONFLICT OF INTEREST

None declared.
